# Traditional radiography versus computed tomography to assess reduced distal radius fractures

**DOI:** 10.1007/s00068-024-02598-5

**Published:** 2024-07-10

**Authors:** Lente H. M. Dankelman, Britt Barvelink, Michael H. J. Verhofstad, Mathieu M. E. Wijffels, Joost W. Colaris

**Affiliations:** 1 Trauma Research Unit, Department of Surgery, Erasmus MC, University Medical Center Rotterdam, 3000 CA, P.O. Box 2040, Rotterdam, The Netherlands; 2 Department of Orthopaedics, Erasmus University Medical Centre, Rotterdam, The Netherlands

**Keywords:** Distal radius fractures, Radiographs, Computed tomography, Malalignment

## Abstract

**Introduction:**

This study compares computed tomography (CT) with plain radiography in its ability to assess distal radius fracture (DRF) malalignment after closed reduction and cast immobilization.

**Methods:**

Malalignment is defined as radiographic fracture alignment beyond threshold values according to the Dutch guideline encompassing angulation, inclination, positive ulnar variance and intra-articular step-off or gap. After identifying 96 patients with correct alignment on initial post-reduction radiographs, we re-assessed alignment on post-reduction CT scans.

**Results:**

Significant discrepancies were found between radiographs and CT scans in all measurement parameters. Notably, intra-articular step-off and gap variations on CT scans led to the reclassification of the majority of cases from correct alignment to malalignment. CT scans showed malalignment in 53% of cases, of which 73% underwent surgery.

**Conclusion:**

When there is doubt about post-reduction alignment based on radiograph imaging, additional CT scanning often reveals malalignment, primarily due to intra-articular incongruency.

## Introduction

Distal radius fractures (DRF) that heal non-anatomically could result in functional impairment in the short term and degenerative changes on the long run. Malalignment of a DRF is defined as radiographic fracture alignment beyond threshold parameters by the Dutch guidelines: ≥ 10° of dorsal angulation, or ≥ 20° of volar angulation, ≤ 15° of inclination, ≥ 3 mm of positive ulnar variance and ≥ 2 mm intra-articular step-off. The question of to which extent fracture displacement can be accepted remains open. Traditionally, fracture displacement is measured on plain radiographs, but the use of computed tomography (CT) scans to guide treatment has increased [1, 2]. A CT scan has the potential to provide more details on the fracture alignment but is less easily available and more expensive, and radiation exposure is increased compared to plain radiographs. Therefore, it is relevant to determine in which specific cases a CT scan adds value to the radiographic parameters used to asses malalignment.

While radiographs are standardly used to determine the existence of a fracture [3–10], additional CT scanning is advised when doubting the alignment or involvement of the articular surface and consequently doubting the necessity for surgical reduction and fixation, according to the Dutch guidelines [11]. Compared to conventional radiographs, additional CT scanning is more accurate in determining the degree of angulation and the involvement of the distal radioulnar joint [4, 12–14]. The treatment choice is adjusted from conservative to operative in 23% to 46% of DRFs after additional CT scanning, and a CT scan improved the intraobserver reproducibility in the choice of surgical treatment [1, 2, 4, 12–20]. However, most of these studies are based on relatively small sample sizes [1, 2, 16, 17, 19–21], evaluated by a limited number of observers, and not evaluating all five fracture characteristics (angulation, inclination, positive ulnar variance, step-off and gap) that are used to guide the treatment modality [11]. Thus, the differences in assessment of all relevant fracture alignment characteristics, measured on radiographs versus CT scans in a large cohort, have yet to be investigated.

The aim of this study is to unravel whether an additional CT scan compared to conventional radiographs will result in different alignment measurements that might cross the border from correct to malaligned in DRFs. In addition, the agreement and reliability between radiographs and CT scans are assessed, with a subanalysis to confound for secondary displacement.

## Methods

### Study population and data selection

According to the local Medical Ethics Committee approved protocol (MEC-2020–0258), cases were selected from a retrospective cohort. This cohort consists of patients who sustained a DRF and were presented at our academic level 1 trauma centre between January 2011 and July 2020. Inclusion criteria were: 1) age ≥ 18 years, 2) reduced DRF, 3) pre- and post-reduction posterior-anterior (PA) and lateral radiographs available, and 4) additional post-reduction CT scans available, taken within seven days after trauma. Exclusion criteria were: 1) no, incomplete or inadequate radiographic follow-up, 2) re-fracture of the distal radius, 3) malalignment post-reduction according to the Dutch Guidelines for DRFs [11], 4) fracture not reduced within 24 h after trauma and 5) initial treatment with external or internal fixation.

### Baseline and fracture characteristics

The following baseline characteristics were collected: age at the time of injury (years), sex, AO fracture classification (A/B/C, according to the trauma radiograph), the interval between trauma and additional CT scanning (days), and – in case of surgical reduction and fixation – the interval between trauma and surgery.

**Fig. 1:**
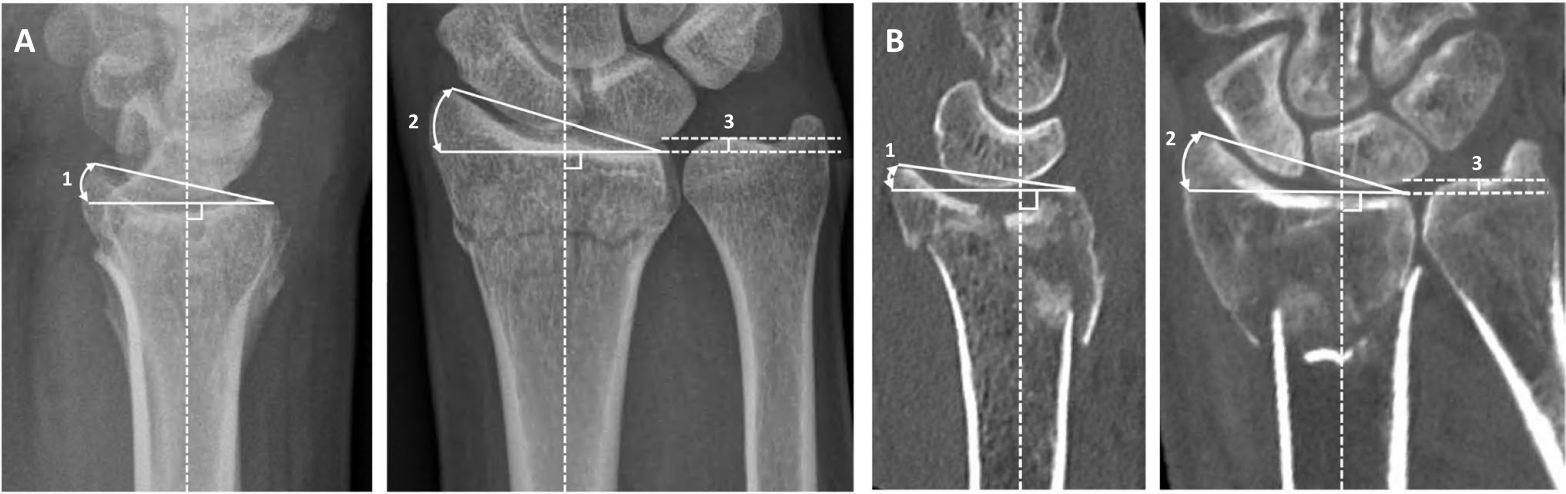
Examples of measurements on radiographs and CT scans: **A** (1) Angulation, (2) inclination, and (3) positive ulnar variance on radiographs; **B** (1) Angulation, (2) inclination, and (3) positive ulnar variance on CT scan

On all radiographs and CT scans, fracture alignment was measured to define whether the fracture was correctly aligned or not. The fracture characteristics that were measured DRFs comprise radial inclination (degrees), positive ulnar variance (mm) and intra-articular step-off and gap (mm) on PA views, and dorsal or volar angulation (degrees) and intra-articular step-off and gap (mm) on lateral views on radiographs (Fig. 1A). Four trained and experienced researchers conducted these alignment measurements according to the same measurement guidelines by Medoff et al. [22]. When doubting measurements, radiographs and CT scans were reviewed by a senior orthopaedic surgeon (JC).

Angulation was measured on the sagittal CT scan, on the slide where the line could be drawn between the uppermost (dorsal and volar) point of the articular surface of the distal radius [23, 24]. Inclination and positive ulnar variance were measured on the coronal CT view, on the slide where the distal-most point of the radial styloid and the midpoint between the dorsal and palmar radial cortical margins was shown [4] (Fig. 1B). Intra-articular step-off or gaps were measured on the slide with the largest step-off or gap [4] (Fig. 1A and B). Measurements were performed using a DICOM viewer, Synedra View Personal, version 20.0.0.4. In case of multiple step-offs or gaps, the largest was described. When a step-off or gap was measured on a CT and could not be seen on radiographs, it was valued as ‘0’ on the radiograph.

### Data presentation

The primary outcomes involved the difference in angulation, inclination, positive ulnar variance, intra-articular step-off, and gap on post-reduction radiographs and additional CT. We assessed each parameter individually to determine if correct alignment or malalignment was seen on CT. Overall, fractures were labelled for all imaging as correctly or malaligned according to the Dutch guideline threshold values. The Dutch guidelines for DRFs state that a fracture is malaligned when one or more of the following threshold values are exceeded: ≥ 10° of dorsal angulation, or ≥ 20° of volar angulation, ≤ 15° of inclination, ≥ 3 mm of positive ulnar variance and ≥ 2 mm intra-articular step-off [11]. The median time between trauma and CT scan and the median time to surgical intervention was calculated. As a secondary outcome, the agreement between the two imaging techniques, radiographs versus CT, and the reliability of the agreement was calculated. In addition, a separate assessment was performed on the subgroup, for which the CT scan was made the same day as the post-reduction radiographs to minimize the change for early secondary displacement.

### Statistical analysis

Data distribution was assessed using the Shapiro–Wilk test, with a p-value < 0.05 indicating non-normal distribution. Missing data were not imputed, and a p-value < 0.05 was deemed significant for all analyses.

Descriptive statistics summarized patient characteristics and radiographic measurements. Continuous data are reported as mean with standard deviation (SD) for normal distributions or median with interquartile range (IQR) for non-normal distributions. Categorical data are presented as counts with percentages.

CT scan analyses included calculating percentages for correct alignment versus malalignment, with 95% confidence intervals (CIs) derived via the modified Wald method. Statistical significance was inferred when the 95% CIs did not encompass 0. The Wilcoxon Signed-Rank test evaluated differences in fracture alignment measured on radiographs and CT scans.

Agreement between imaging methods and their clinical relevance was examined through Bland–Altman analysis, plotting mean measurements against their differences. Points scattered above 0 with a 95% CI above 0 indicated that the CT scan measurements were larger than those on radiographs. This analysis, along with Intra Class Correlation (ICC) for reliability (categorized by Koo and Li, 2016 as “poor” < 0.5, “moderate” 0.5–0.75, “good” 0.75–0.90, and “excellent” > 0.90), highlighted systematic biases and agreement levels. Both analyses extended to acute CT scans.

## Results

### Study population

**Fig. 2:**
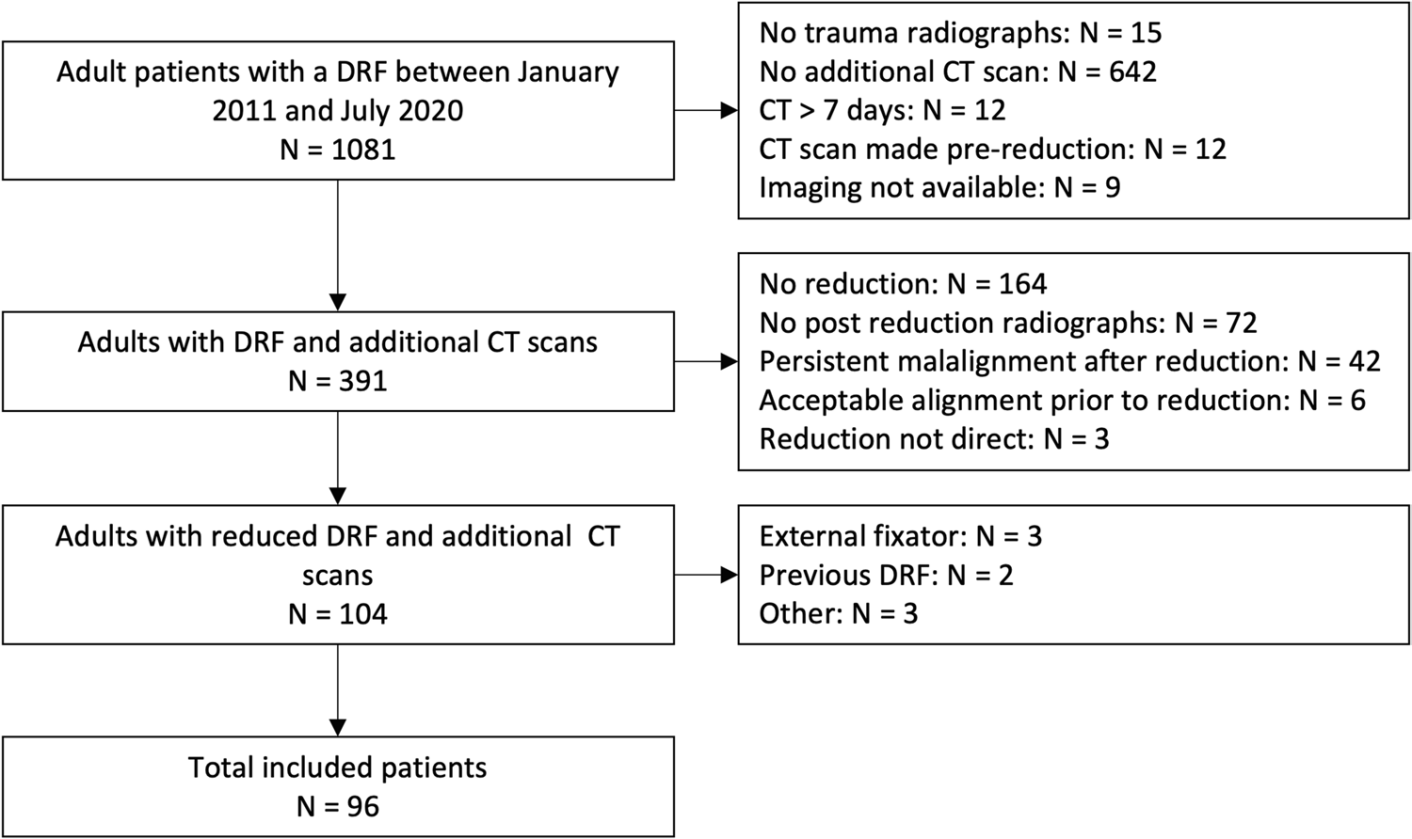
Flowchart of the study population. DRF: Distal radius fracture; CT: Computed tomography

We included 96 patients with 96 DRFs from our database from 2011 to 2020. A flowchart of the inclusion process is shown in Fig. 2. Baseline characteristics are demonstrated in Table 1. According to the AO classification, most fractures concern AO type C (68%). The median interval from presentation at the emergency room to CT scanning was three days (IQR 2–4 days), and in 55% of cases, the CT scan was performed on the day of reduction. A total of 63 patients (66%) were treated surgically; the median time from trauma to surgical fixation was five days (IQR 3–9 days).

**Table 1:** Baseline patient- and fracture characteristics

Characteristic	*n* = 96
Age in years, mean (SD)	52 (SD 17.5)
Female, *n* (%)	60 (63%)
AO classification, *n* (%)	
A Extra-articular	19 (20%)
B Partial articular	12 (12%)
C Complete articular	65 (68%)

### Primary outcome

The median measurements on all parameters differed significantly when comparing radiographs and CT scans (Table 2). Radiograph measurements and CT scans agreed that after reduction, 68 (71%) of DRFs were dorsally angulated and 28 (29%) volarly. In contrast with the acceptable sagittal angulation on radiographs, measurement on CT scan revealed unacceptably dorsal angulation in 20 (29%) patients and unacceptably volar angulation in 3 (11%) patients. Inclination was measured in all cases, revealing malalignment in 17 cases (18%), only indicated by CT scans. Positive ulnar variance was measured in 36 (38%) cases, of which in 3 (8%) cases, a positive ulnar variance ≥ 3 mm was measured on CT only. An intra-articular step-off or gap was measured in 28 (29%) and 76 (79%) DRFs, respectively, resulting in malalignment in 20 (71%) and 69 (91%) of the cases on CT.

**Table 2:** Fracture alignment measured on post-reduction radiographs and CT

	Cases *n* = 96	Post-reduction alignment on radiographs	Post-reduction alignment on CT-scan	*P*-value^b^	Malalignment conform CT imaging^c^
Angulation					
Dorsal, °	68^a^	5.0 (2.0–8.0)	6.0 (3.5–11.0)	**0.002**	20 (29%, 0.19–0.42)^d^
Volar, °	28^a^	6.0 (1.0–10.8)	8.5 (3.3–14.0)	**0.015**	3 (11%, 0.03–0.28)^d^
Inclination, °	96^a^	22.0 (18.0–23.7)	20.5 (17.0–23.0)	**0.016**	17 (18%, 0.11–0.27)^d^
Positive ulnar variance, mm	36^a^	1.7 (1.0–2.0)	2.1 (1.5–2.4)	**0.007**	3 (8%, 0.05–0.30)^d^
Step-off, mm	28^a^	1.0 (0.0–1.6)	2.1 (1.5–2.3)	** < 0.001**	20 (71%, 0.51–0.86)^d^
Gap, mm	76^a^	1.4 (1.0–2.0)	4.1(2.4–5.8)	** < 0.001**	69 (91%, 0.81–0.95)^d^

If not noted differently, information is presented as median with interquartile range between parentheses

^a^Includes the number of DRFs in which this fracture parameter was measured

^b^Wilcoxon Rank Sum test was used

^c^Number of cases in which fracture alignment was not acceptably aligned on the CT scan

^d^The 95% confidence intervals were calculated using the modified Wald method

In 53% of cases, the fracture was labelled correctly aligned on post-reduction radiographs, while additional CT scanning revealed a malalignment. In the other cases (47%), there was an agreement on both radiographs and CT scans on correct fracture alignment. Divided by fracture morphologies according to the AO classification (A/B/C), CT scans revealed malalignment in 52%, 41%, and 58% of the cases, respectively. The DRF was surgically treated in 73% of cases in which radiographs and CT disagreed. The remaining 27% with malalignment conform CT scan measurements were conservatively treated. In addition, surgery was chosen in 13% of DRFs that were correctly aligned conform the guideline. The number needed to treat (NNT), or as in this study, 'the number needed to diagnose', was 1.89. This indicated that approximately two patients would need to be assessed using CT scans instead of radiographs to correctly identify one additional case of malalignment that was misdiagnosed by radiographs.

### Secondary outcome

**Fig. 3:**
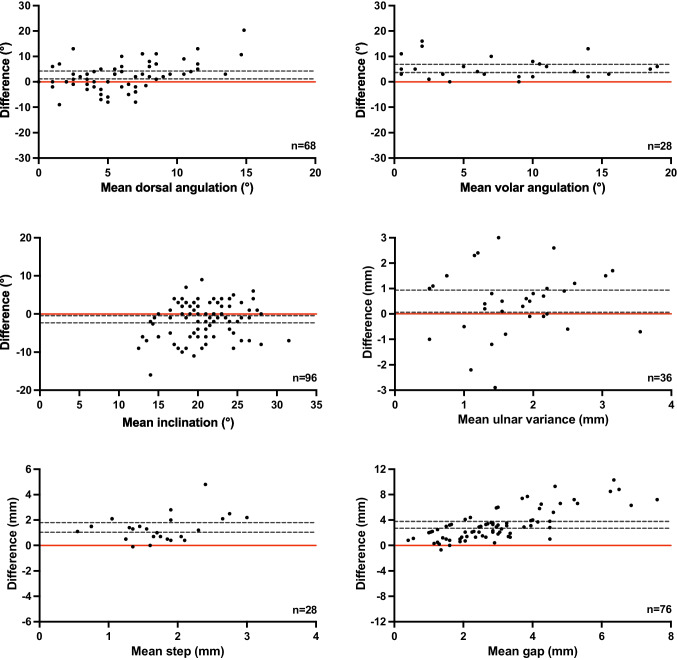
Bland–Altman Plots of the differences between radiographs versus CT scans. The CT measurements were subtracted from radiograph measurements. Horizontal black lines display the limits of agreement (95% CI). Points scattered above 0 with a 95% CI above 0 (red line) indicated that the measurements on the CT scan were larger than the measurements on radiographs

The agreement and reliability for all measurements between radiographs and CT scans were calculated (Table 3). Figure 3 shows the Bland–Altman plots assessing the agreement, showing that the differences vary systematically for all measurements. CT scans showed significantly increased angulation severity, loss of inclination, positive ulnar variances and intra-articular incongruences (Fig. 3).

**Table 3:** Correlation between alignment measurements performed on radiographs versus CT-scan

	Cases *n* = 96	Intraclass Correlation^b^	95% Confidence Interval	Reliability
Angulation, °				
Dorsal	68^a^	0.19	-0.03–0.40	Poor
Volar	28^a^	0.35	0.01–0.63	Poor
Inclination, °	96^a^	0.44	0.26–0.59	Poor
Positive ulnar variance, mm	36^a^	0.05	-0.23–0.34	Poor
Step-off, mm	28^a^	0.11	-0.09–0.37	Poor
Gap, mm	76^a^	0.09	-0.07–0.27	Poor

^a^Includes the number of DRFs in which this fracture parameter was measured

^b^Type A intraclass correlation coefficients using an absolute agreement definition. Values < 0.5 indicates poor reliability

The intraclass correlation (ICC), which indicates reliability between the two imaging techniques, showed that the radiographs and CT scans were in poor agreement for all alignment measurements. The ICC also showed poor reliability for all measurements (Table 3).

**Fig. 4:**
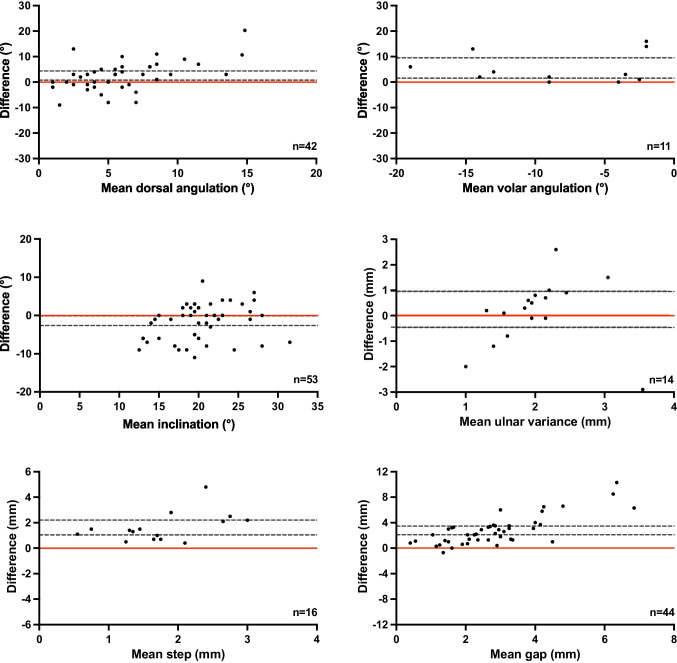
Sub analysis of radiographs versus CT scans made on the same day. Bland–Altman Plots of the differences between radiographs versus CT scans. The CT measurements were subtracted from radiograph measurements. Horizontal black lines display the limits of agreement (95% CI). Points scattered above 0 with a 95% CI above 0 (red line) indicated that the measurements on the CT scan were larger than the measurements on radiographs

In 55% of the included cases, the CT scan was obtained immediately after reduction. The separate ICC and Bland–Altman analysis for these cases showed differences in angulation, inclination, step-off and gap measurements. Only measurements of positive ulnar variance showed a negative ICC and did not vary systematically on the Bland–Altman plots (Table 4 and Fig. 4).

**Table 4:** Sub analysis for correlation between alignment measurements on radiographs versus CT-scan, performed on same day

	Cases *N* = 53	Intraclass Correlation^b^	95% Confidence Interval	Reliability
Angulation, °				
Dorsal	42^a^	0.15	-0.12–0.42	Poor
Volar	11^a^	0.27	-0.16–0.69	Poor
Inclination, °	53^a^	0.52	0.30–0.69	Poor
Ulnar positive variance, mm	14^a^	-0.03	-0.27–0.30	Poor
Step-off, mm	16^a^	0.11	-0.10–0.43	Poor
Gap, mm	44^a^	0.11	-0.08–0.33	Poor

^a^Includes the number of DRFs in which this fracture parameter was measured

^b^Type A intraclass correlation coefficients using an absolute agreement definition. Values < 0.5 indicates poor reliability

## Discussion

This study shows that conventional radiographs consistently underestimate reduced DRFs’ severity compared to CT scans based on volar and dorsal angulation, loss of inclination, positive ulnar variance and intra-articular incongruence. In 53% of cases, additional CT scanning showed malalignment, while they appeared correctly aligned based on radiograph measurements. The ICC and Bland-Altmant plots showed a clear discrepancy between the two imaging techniques on all measurement parameters, whereas CT scans showed significantly increased severity on all alignment measurements compared to radiographs.

In line with our findings, previous studies reported that radiographs tend to underestimate intra-articular incongruence concluding that the CT scan is more reliable for the measurement of intra-articular involvement in DRFs [4, 12–15]. Furthermore, previous research has shown that CT scans increase inter-surgeon agreement on the need for surgical intervention [2, 14–18]. Additional CT scanning changes the indication from conservative treatment to surgery in 23% to 46% of cases [2, 15, 19, 21]. Therefore, in cases of uncertainty regarding the alignment after reduction, especially concerning the intra-articular incongruence, a CT scan may offer additional value. Future studies need to asses if this consideration would contribute to eventually improved clinical outcomes.

Although the Dutch guideline for DRFs advises operative treatment for malaligned fractures [11], approximately a quarter of malaligned DRFs in this cohort were treated conservatively. Potential reasons can be patient-related (e.g., age or concomitant health problems being a risk for surgery in general), fracture-related (e.g., alignment was close to threshold values), or surgeon-related (e.g., reluctance to operate on severely comminuted fractures). Due to the retrospective nature of this study, the exact reasons for the chosen treatment modality remain unknown.

Before advocating surgical intervention to prevent malunion, one has to realize that previous studies showed a poor correlation between malunion and clinical outcomes, especially in older patients [11]. Studies report malunion rates of 35% in non-surgically treated fractures and 10% in surgically treated fractures [25, 26]. Malunion might result in chronic pain, reduced function, decreased grip strength and impaired ability to perform daily activities [27–29]. Secondary invalidating osteoarthritis can also be initiated due to uneven force distribution across the radiocarpal joint surface [30]. Further studies are needed to accurately determine the level of malalignment that leads to clinically unacceptable outcomes.

We decided to define the acceptability of fracture alignment conform the Dutch guidelines for DRFs. Simply because retrospective cases were used that were treated conform this guideline. Secondly, the Dutch guideline comprises a broad assessment of alignment. Volar angulation and inclination are not encountered in the American Academy of Orthopaedic Surgeons guidelines [31]. However, both guidelines agree on the threshold values for dorsal angulation, positive ulnar variance, and step-off or gap. Our analysis revealed that shifts from correct to malalignment primarily occurred in measurements step-off or gap, parameters recognized by both guidelines.

This study needs interpretation in light of its strengths and limitations. To date, this study is the first to evaluate all these characteristics on radiographs and CT scans based on a large cohort of DRFs. Previous studies either only assessed intra-articular involvement [4, 14, 15] or only the extra-articular radiographic parameters [17]. Furthermore, we consciously chose only to include cases in which the CT scan was made shortly after (within seven days) reduction. Additionally, the subgroup analysis on cases where the CT scan was performed immediately after reduction, which minimized the risk of secondary displacement, showed similar results. Therefore, it can be concluded that the discrepancies between the radiograph and CT are not attributed to secondary displacement.

As the first limitation, there was a potential for selection bias. According to the guidelines, a CT scan is made when doubting the alignment of a DRF and for pre-operative planning. Due to the retrospective design of this study, the exact reason behind the physician's decision to perform a CT scan is unknown. Therefore, conclusions should be carefully interpreted and are only applicable on cases in which post-reduction fracture alignment is doubted. Secondly, the measurements were not repeatedly executed by different observers, which might have resulted in undetected measurement errors. Consequently, inter- and intra-observer reliability of measurements is not presented. However, Watson et al. showed that the intra-observer reliability is high for angulation measurements and moderate for inclination and positive ulnar variance measurements on radiographs [32]. Lastly, in some cases, it was difficult to determine the axis of the radius on CT scans due to the truncation of the radial shaft. This might have influenced the angulation and inclination measurements since these are based on the radial shaft axis. However, the suboptimal radiology results depict more of the daily clinical situation than the optimal scientific situation, enabling extrapolation of the results.

This study suggests that additional CT scanning often shows DRF malalignment. According to our findings, the differences between radiographs and CT scans on step-off and gap measurements might have clinical implications because these measurements appeared beyond the guideline’s threshold in 71% and 91% of the cases, respectively. In patients with any uncertainty about the articular congruency, a CT scan can provide valuable insights into fracture alignment. Therefore, a CT scan might help to plan a surgical approach. However, it is essential to consider the additional costs and the radiation exposure associated with additional CT scans, while the clinical impact remains unknown. Future research should assess the cost-benefits of additional CT scans of reduced DRFs. Furthermore, it should be taken into account that DRF treatment is not only based on radiological parameters. More aspects of the patient's condition and preferences should be considered when deciding on the optimal treatment for a DRF.

In conclusion, our study consistently demonstrates an underestimation of DRF alignment on radiographs compared to CT scans. According to the guideline, this leads to a shift from correct alignment to malalignment in over half of the cases, mainly underestimating intra-articular step-off and gap measurements. Our finding emphasizes the clinical significance of incorporating CT scans in evaluating and managing displaced DRFs in which post-reduction alignment is doubted. Further evaluation is needed to assess the effect of the implications of these findings, and it is essential to extend our focus on the importance of patient preferences beyond radiographic parameters.

## Data Availability

No datasets were generated or analysed during the current study.
